# A Novel Light‐Induced Collective Circular Movement in *Armadillo sordidus* Isopods

**DOI:** 10.1002/ece3.73487

**Published:** 2026-04-13

**Authors:** Idan Sheizaf, Eviatar Itzkovich, Ariel D. Chipman

**Affiliations:** ^1^ Department of Ecology, Evolution and Behavior, the Silberman Institute of Life Sciences The Hebrew University of Jerusalem Jerusalem Israel; ^2^ Amateur Naturalist Kibbutz Gshur Israel

**Keywords:** aggregative behavior, isopod, light pollution, movement

## Abstract

Collective movement in terrestrial isopods has rarely been documented and almost never discussed. Here, we report a novel behavioral phenomenon in isopods from the species *Armadillo sordidus* : large nocturnal aggregations forming coordinated circular movements involving thousands of individuals. The behavior was observed naturally in several locations in northern Israel and could be experimentally induced using artificial white light. Ultraviolet light and magnetic fields did not induce this behavior. Image analysis revealed approximately 5500 individuals within a single aggregation, and manual tracking confirmed a clear circular movement pattern. The observed sex ratio (1:4 males to females) and presence of many gravid females suggest that the behavior is not reproductive. Instead, the circular motion appears to represent a density‐dependent, light‐induced aggregation response. To our knowledge, this is the first formal documentation of collective circular movement in isopods, potentially arising from anthropogenic light pollution.

## Graphical abstract

We describe an interesting aggregative behavior in terrestrial isopods. We conclude that this behavior is in response to intense artificial lighting.

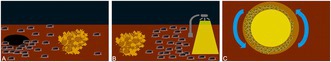

## 1 Introduction

Collective mass movement is a remarkable phenomenon seen across the animal kingdom, from mammals like the blue wildebeest ( *Connochaetes taurinus* ) (Torney et al. 2018) and the caribou ( *Rangifer tarandus granti* ) (Joly 2025), to arthropods such as the monarch butterfly ( *Danaus plexippus* ) (Reppert and de Roode 2018) and Locusts (e.g., *Locusta migratoria*) (Ariel and Ayali 2015). The reasons are varied and revolve around the idea of strength in numbers, which benefits the group as well as the individual. Benefits include enhancing navigation, avoiding predation, improving reproduction, foraging, and more. This type of movement often arises in response to an environmental challenge, such as increased population density, shortage in food supply, inadequate environmental conditions, and similar issues, which may be resolved by improving the surroundings (Bode et al. 2010; del Mar Delgado et al. 2018; Shellard and Mayor 2020). However, these studies usually deal with the effects of mass vectorial movements (migration), movements which start at a start‐point and end at a destination. Beyond migration, some animals perform non‐migratory collective movements, like army ants ( *Eciton burchellii* ) (Reynolds et al. 2015) raiding for food, and fish schooling for defense (Pavlov and Kasumyan 2000). Together, these diverse phenomena highlight how simple social rules and environmental pressures can generate complex, large‐scale coordination in animal movement.

While collective movements often confer adaptive benefits, some arise without clear advantages and may even prove detrimental. Well‐known examples of this include the circular milling behavior in army ants ( *E. burchellii* ), where looping pheromone trails in featureless environments cause continuous circular marches, a phenomenon similarly observed in the highly coordinated, nose‐to‐tail processions of the pine processionary caterpillar (*Thaumetopoea pityocampa*), and others (Delcourt et al. 2016; Schneirla 1944). Similarly, external factors in modern environments can provoke unintended synchronous behaviors; artificial light at night (ALAN), for example, attracts and disorients nocturnal arthropods like butterflies and beetles, prompting erratic mass clustering or orbiting around lamps that hinders mating and foraging, while disrupting natural cues and reducing survival (Owens and Lewis 2018; Foster et al. 2021). These disruptions highlight the importance of studying how human‐induced changes interfere with innate movement synchronization, within broader ecological research.

Magnetism is an additional factor that may profoundly influence animal locomotory behavior across diverse taxa. Magnetoreception enables organisms to detect Earth's magnetic field for navigation and orientation. Birds are among the best‐studied examples, using magnetic field information as both a directional compass and navigational map during long‐distance migration through cryptochrome proteins in the eye and magnetite‐based receptors in their beaks (Cadiou and McNaughton 2010; Wiltschko and Wiltschko 2019). Magnetoreception extends to arthropods, where honeybees ( *Apis mellifera* ) use geomagnetic information during waggle dances and foraging, while various ant species (e.g., *Myrmica ruginodis* , *Oecophylla smaragdina* ) orient their path‐integrated navigation using a magnetic compass, with some aligning seasonal migrations along the magnetic North–South axis ( *Pachycondyla marginata* ) (Wajnberg et al. 2010). Non‐insect crustaceans also demonstrate these abilities: Caribbean spiny lobsters ( *Panulirus argus* ) use Earth's magnetic field as a navigational cue with magnetite‐based receptors (Ernst and Lohmann 2016). Crayfish ( *Cambarus appalachiensis* ) display spontaneous magnetic alignment along the northeast‐southwest axis influenced by their ectosymbiotic branchiobdellidan worm densities (Landler et al. 2019). Velvet crabs (*Necora puber*) show behavioral responses to artificial magnetic fields (Albert et al. 2023). These insects and crustaceans possess magnetic nanoparticles that likely facilitate magnetoreception, using the earth's magnetic field as a compass against which other orientation cues are calibrated.

The Golan Heights, in Northern Israel, have distinctive magnetic field properties that could affect how animals navigate and orient themselves in this region (Shnaidman et al. 2017). The magnetic field strength in northern Israel, including the Golan Heights, is approximately 44,600 nanoTesla—about 4.5% stronger than in southern Israel (42,700 nanoTesla) (Shnaidman et al. 2017)—a difference that magnetically sensitive animals may be able to detect. The Golan Heights is a volcanic plateau formed by Plio‐Pleistocene lava flows, and these basalt rocks create local areas where the magnetic field is “flipped”—pointing in the opposite direction to Earth's current field (Frank et al. 2002; Behar et al. 2019). The magnetic field direction in the Golan Heights has also shifted over time, with the angle between magnetic north and true north currently at about 4.85° East, having changed by roughly 5° over the past century (Shnaidman et al. 2017; Frank et al. 2002; Behar et al. 2019). These variations in field strength, shifting magnetic north, and local magnetic anomalies could all influence how animals that use magnetoreception—such as birds, insects, and crustaceans—navigate through or live in this region.

The crustacean order Isopoda exhibits remarkable ecological diversity, with species occupying habitats ranging from the deep sea to terrestrial environments and across a wide range of climatic conditions, from polar regions to arid deserts (Poore and Bruce 2012; Wilson 2008; Sfenthourakis and Hornung 2018). The suborder Oniscidea, commonly known as woodlice, constitutes a monophyletic group comprising all terrestrial isopods and represents the most species‐rich lineage of terrestrial crustaceans (Sfenthourakis and Hornung 2018; Thomas Thorpe 2024). As key detritivores, they play a crucial ecological role by decomposing organic matter such as leaf litter, animal carcasses, and fecal material (Zimmer 2002).

Terrestrial isopod behavior is strongly influenced by moisture requirements, typically resulting in nocturnal activity and diurnal aggregation under rocks, logs, or debris to avoid desiccation (Hornung 2011; Leclercq‐Dransart et al. 2019; Delhoumi et al. 2023). While mass aggregations are well‐documented as a desiccation‐avoidance strategy, reports of large‐scale, directional, or coordinated surface movements are rare and largely anecdotal.

The few recorded instances of aggregative behavior in isopods—summarized by Warburg (Warburg et al. 1984; Warburg 1993) describe both identified and unidentified species (*Porcellio* sp., *Buddelundia* sp., *Hemilepistus reaumuri* , *Porcellionides pruinosus* , and *Porcellio scaber* ), yet none provide quantitative or behavioral analyses and describe a massive vectorial movement toward an unknown destination. Warburg (Warburg et al. 1984; Warburg 1993) describes his observations of massive aggregative behavior of isopods, always in chance observations and never well‐studied. In his book (Warburg 1993) (Chapter 12), Warburg writes that the phenomenon has never been studied and …“The solution will have to await a detailed study”. Unfortunately, Warburg never conducted such a study, nor did anyone else. The lack of detailed documentation leaves a gap in our understanding of collective behavior in terrestrial isopods.

Here, we describe a novel and striking behavioral phenomenon, observed by chance, by amateur naturalists, which entails massive aggregations of *Armadillo sordidus* isopods; large‐scale, circular movement occurring during summer nights in two sites in northern Israel. We hypothesize that this behavior represents an unrecognized form of collective response, potentially influenced by environmental or anthropogenic factors, and specifically test for the involvement of two, non‐exclusive mechanisms: (1) geomagnetic anomalies, characteristic of the Golan Heights disrupt or influence isopod orientation, promoting collective circular motion and (2) ALAN from nearby streetlights elicit or modulate collective surface activity, either by attracting individuals, or by interfering with their natural orientation cues. The phenomenon bears a superficial resemblance to the “ant mill” observed in army ants—where individuals form a continuous circular procession until exhaustion or death (Schneirla 1944).

## 2 Methods

### 2.1 Study Sites and Documentation

**FIGURE 1:**
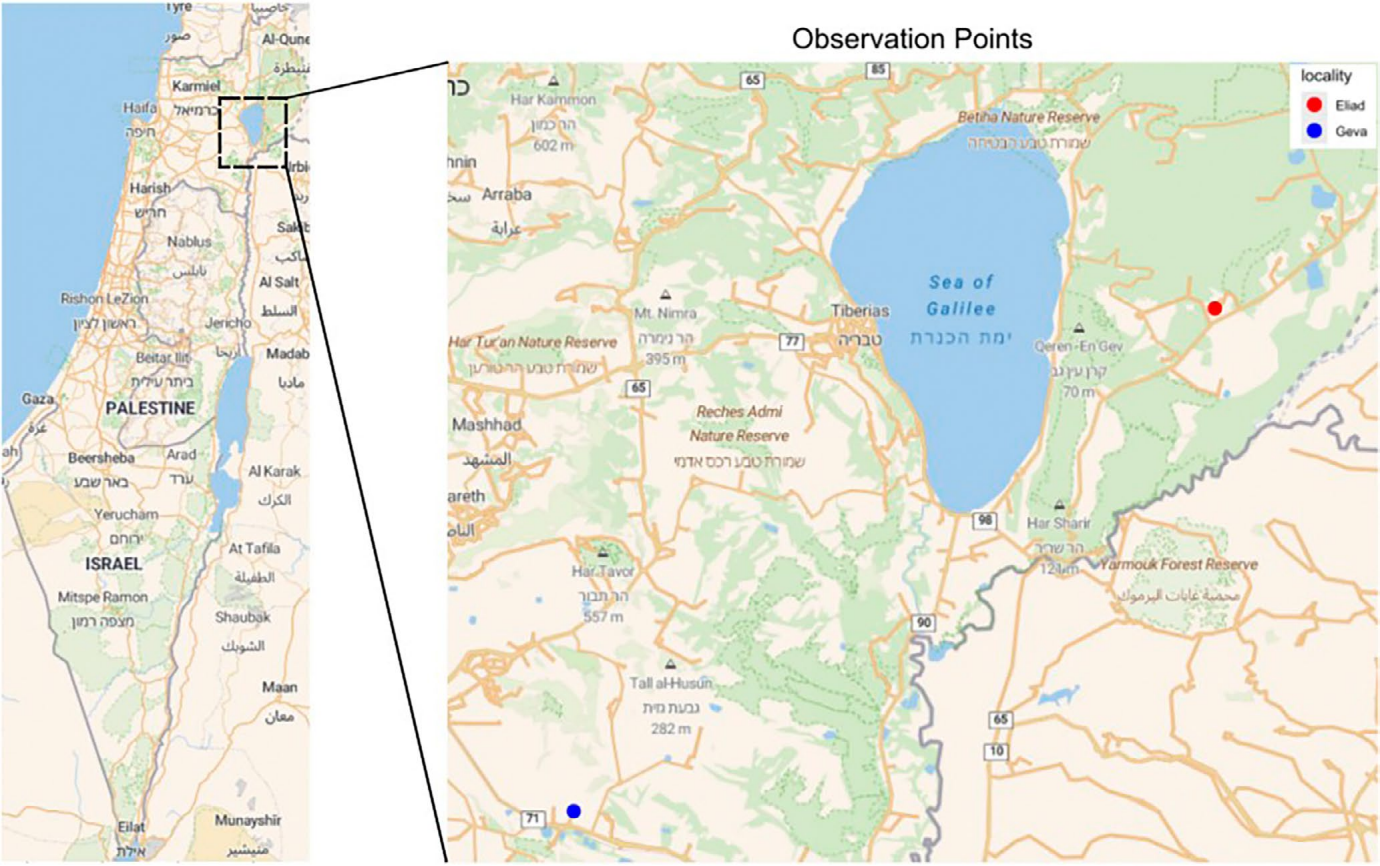
A map of the general locations in which the phenomenon was reported. The map was generated using R (R Core Team 2025) with the packages ggmap (Kahle and Wickham 2013), osmdata (Padgham et al. 2017), tidyverse (Wickham et al. 2019), and Polychrome (Coombes et al. 2019). Code is provided in the Supporting Information.

Chance observations of the circular movement behavior in isopods were reported by amateur naturalists from two locations in Israel (Figure 1):

1. Geva, Jezreel Valley (32°33′54.1″ N, 35°22′16.6″ E), approximately 8 km southeast of Afula.
2. Eliad, southern Golan Heights (32°48′05.8″ N, 35°43′51.3″ E), approximately 8 km east of the Sea of Galilee.

Naturally occurring behavior was documented in the field. Videos and photographs were recorded using standard smartphone cameras (Samsung Galaxy A31, default camera application). Filming occurred exclusively at night during observed activity periods, in the summer, when the sky was clear, the illuminated surface of the moon was between 6% and 91%, and the temperature was between 23°C and 29°C.

### 2.2 Experimental Manipulations

To examine potential drivers of the circular movement behavior, we attempted to induce this behavior via experimental manipulation, in one of the sites where this behavior was observed (Eliad in the Golan Heights). We employed three experimental manipulations to test three hypotheses for the driving force behind this behavior. First, a strong magnet was positioned at the periphery of an active circle (~0.5 m from the rim) to test for responses to magnetic disturbance. Second, a Lumitec white‐light emergency lamp (22 W max power, 6000 K color temperature, 44 × 0.5 W SMD LEDs) was placed parallel or perpendicular to the ground to assess phototactic effects. Third, a Superfire UV06 ultraviolet flashlight (395 nm wavelength, 6 W power, powered by 6× AA batteries) was mounted on a 1 m tall tripod ~5 m from aggregated isopods when oriented perpendicular to the ground or placed at ground level when oriented parallel to the ground. Each light source was left on for 10 min during active nighttime periods.

### 2.3 Specimen Collection and Rearing

Approximately 100 individuals were collected by hand from the Eliad site on August 12, 2025, and brought to the lab at the Hebrew University of Jerusalem. Specimens were maintained in aerated plastic containers containing damp soil and dry oak leaves for documentation, species identification and sex determination. They were reared with a 14 h light and 10 h dark cycle, at a temperature of 23°C and humidity fluctuating between 30% and 70%. After analyses, the majority of them were released back to their original habitat, with two deposited as voucher specimens in the National Natural History Collections at The Hebrew University (Catalog numbers: 0000101262, 0000101263).

### 2.4 Specimen Photography

Specimens brought to the lab were photographed at high resolution using a Nikon SMZ25 microscope fitted with a Nikon Fi3 camera driven by NIS Elements 5.21.03 software. Between 10 and 100 focal planes were taken per specimen and combined using Zerene Stacker 1.04 (PMax protocol, default parameters) for clear species identification.

### 2.5 Isopod Counting Analysis

Isopod abundance was quantified from still frames from the video documenting the behavior in nature, using ImageJ v1.54p4 (Schindelin et al. 2012). A clear video frame was converted to 8‐bit grayscale, inverted, and background‐subtracted (rolling ball radius = 50). A calibrated grid of 88 rectangles was overlaid for spatial segmentation. Thresholding (Li filter) isolated individual isopods as discrete particles. Counts were obtained from each grid cell using *Analyze Particles* (size = 1–7 pixels), and results were summed across all cells. Output data were exported as CSV files for statistical analysis.

### 2.6 Tracking Analysis

Individual trajectories of isopods performing circular movement were manually tracked in ImageJ v1.54p4 using the MTrackJ plugin (Meijering et al. 2012). Videos were first stabilized using the Image Stabilizer plugin (5) with *Transformation = Translation*. After cropping and 3× spatial scaling, 60 visually distinguishable isopods were tracked throughout their visible duration. Tracks were exported as uncompressed AVI videos for visualization and analysis.

## 3 Results

**FIGURE 2:**
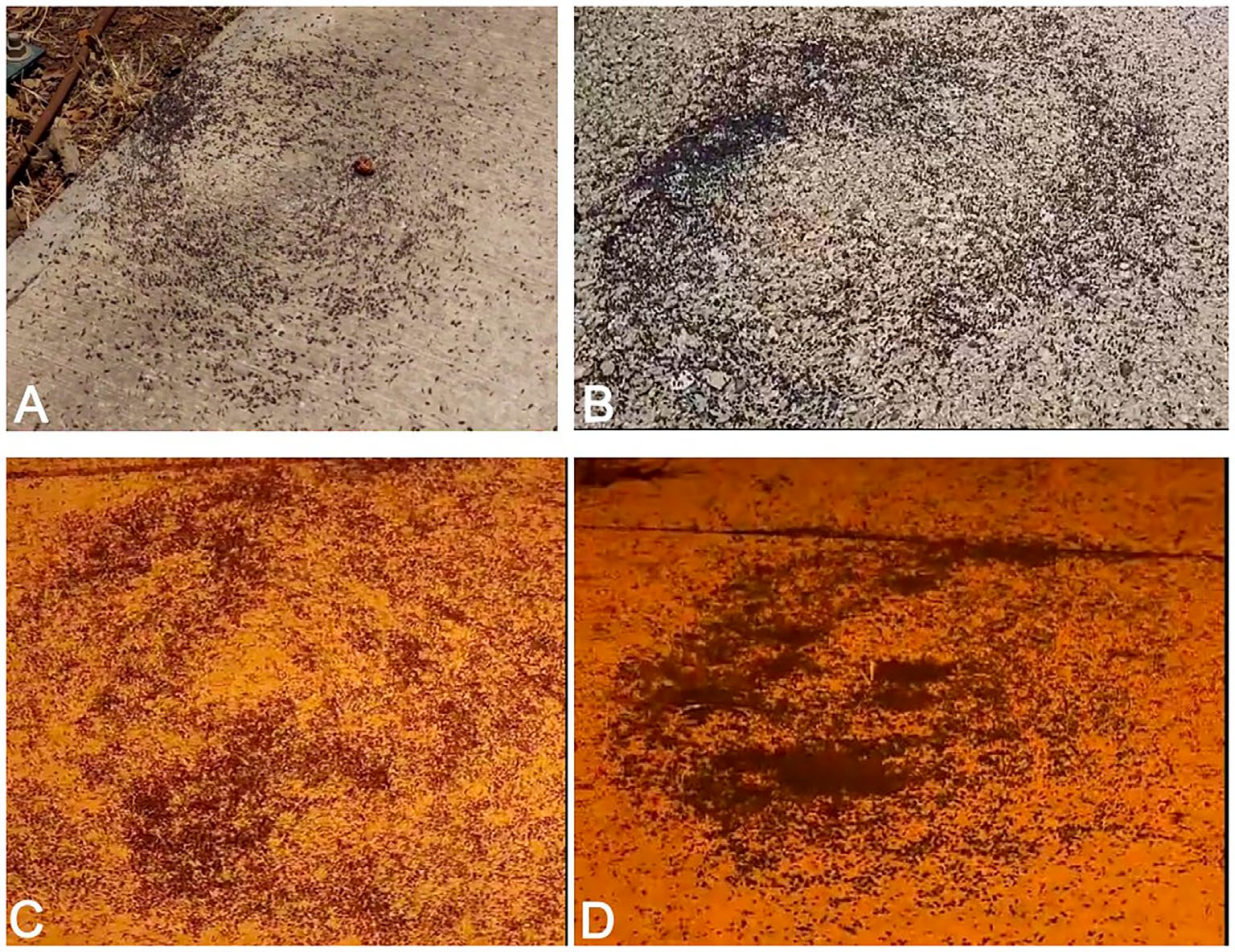
Captured frames from videos showing the circular motion and aggregations from the various locations and dates in Israel. (A) Video S1, captured on July 7, 2021, showing aggregative circular motion from Geva. (B) Video S2, captured on August 13, 2023, showing aggregative circular motion from Eliad. (C) Video S4, captured on August 11, 2025, showing aggregative circular motion from Eliad. (D) Video S5, captured on August 16, 2025, showing aggregative circular motion in Eliad.

Analysis of five available videos (Videos S1, S2, S3, S4, and S5) revealed consistent patterns across five nighttime observations of *A. sordidus* isopod aggregations, spanning 2021–2025 in the Jezreel Valley, and in Eliad in the Golan Heights. In four videos (July 7, 2021; August 13, 2023; August 11 and 16, 2025), thousands to tens of thousands of healthy, active isopods formed pronounced circles—either clockwise or counterclockwise—around an isopod‐free center on gravelly soil or concrete pavement (Figure 2A–D; Videos S1, S2, S4, and S5). This phenomenon was recorded exclusively at night, near active streetlights. The remaining video (August 6, 2025) showed a massive aggregation exhibiting multidirectional movement, without a clear circular pattern, on a concrete surface (Video S3).

### 3.1 Sex Ratio and Species Identification

We collected 102 individuals from the epicenter of a circular aggregation event, yielding a sex ratio of 20 males to 82 females (1:4.1), with 24 gravid females among them (gravid: non‐gravid ratio = 1:3.4).

**FIGURE 3:**
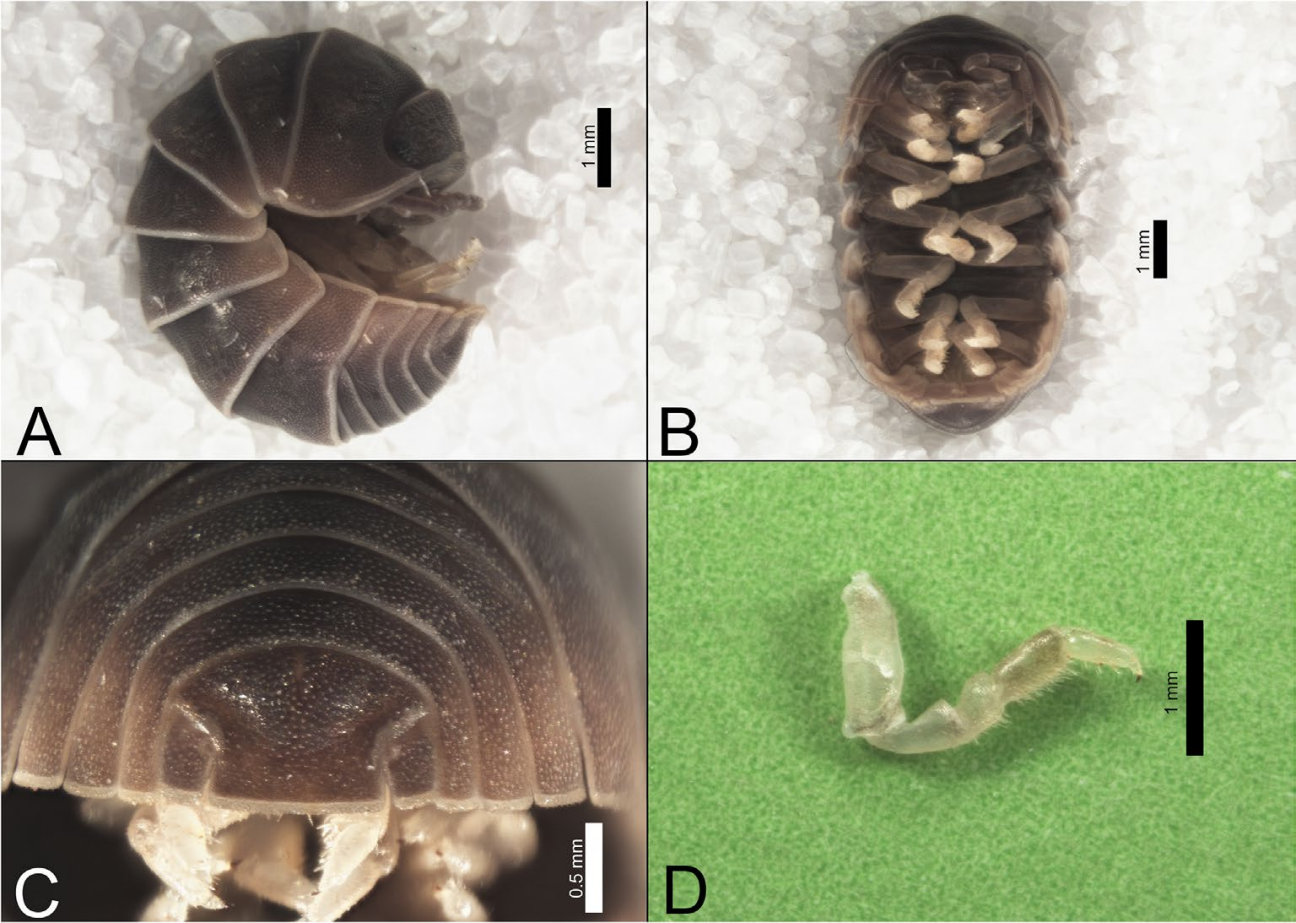
Habitus of isopods collected during aggregative behavior in Eliad. (A) Conglobated lateral view. (B) Relaxed ventral view. (C) Posterior view of the telson. (D) Dissected male Pleopod VII.

Species identification followed Schmalfuss (1996), with five specimens we examined giving the best match to *A. sordidus* . Diagnostic apomorphies included a length‐width ratio of ~3:2, a multi‐pointed setae brush on male Carpus VII, and the shape of the telson's apical part (Figure 3). The collection location matched the species' reported distribution, and the relatively small adult size was also consistent with Schmalfuss's description.

### 3.2 Induction of Mass Circular Movement

We tested our aforementioned hypotheses using three experimental approaches all conducted in Eliad during naturally occurring aggregative circular movements. First, we assessed the role of magnetic disturbance by placing a strong magnet in close proximity (~0.5 m) to an active aggregation; isopods showed no detectable behavioral response and maintained their normal circular movement pattern (Videos S6 and S7).

**FIGURE 4:**
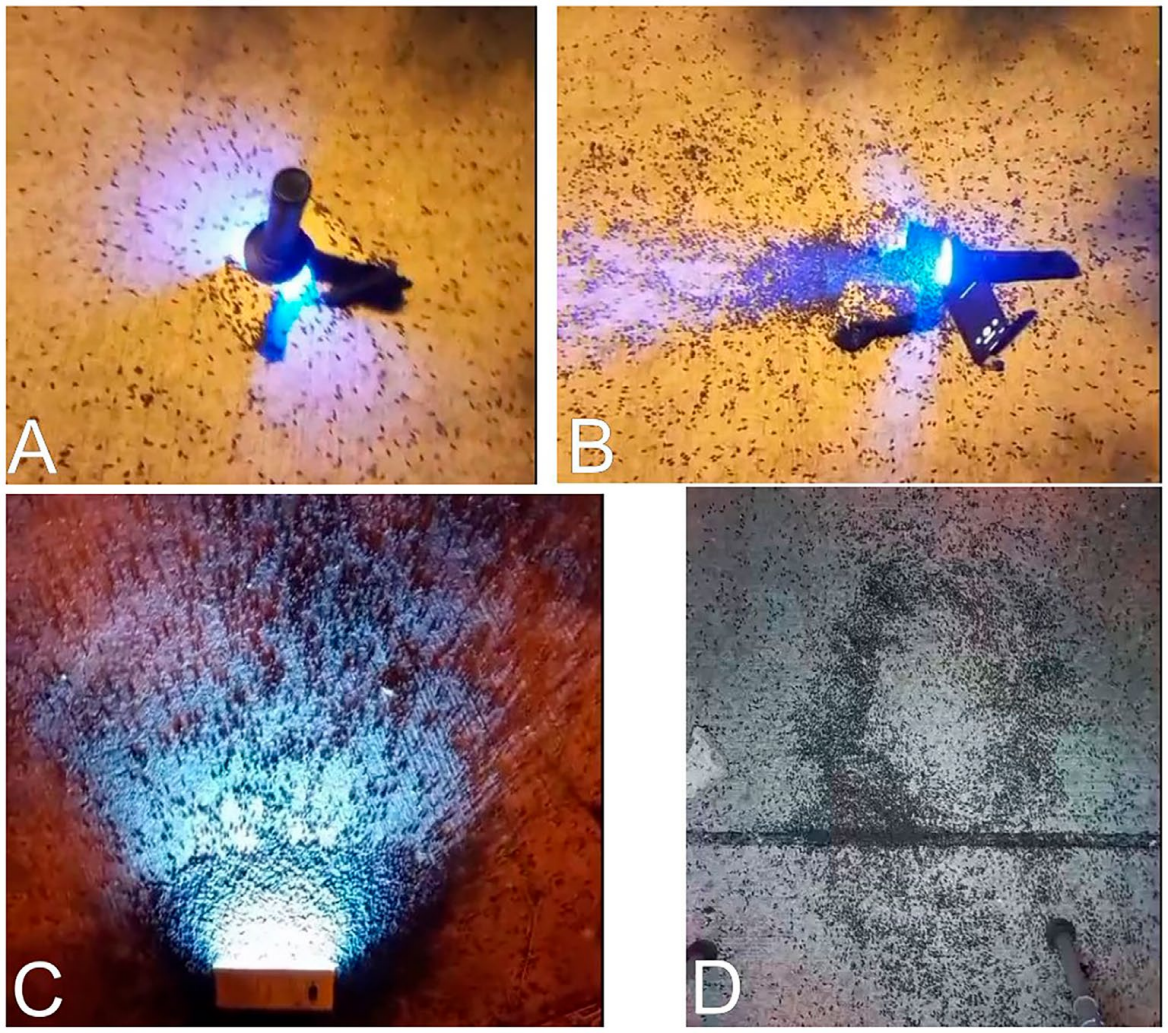
Experiments performed in Eliad. (A) UV light perpendicular to the ground, with few isopods aggregating around it. (B) UV light in parallel to the ground, with some isopods aggregating near it. (C) White light in parallel to the ground, with thousands of isopods aggregating in very close proximity to the light source. (D) White light perpendicular to the ground, after thousands of isopods migrated toward the center of the beam and started performing circular motion around it.

Second, we examined responses to ultraviolet light using a UV flashlight (395 nm, 6 W) positioned at two orientations. When placed parallel to the ground at a distance of ~5 m from the aggregation, or on a 1 m high tripod, and left undisturbed for 10 min, isopods moved toward the light source but did not establish circular motion (Figure 4A; Videos S8 and S9). When the UV light was repositioned parallel to the ground at ground level for 10 min, approximately 5%–10% of individuals were attracted to it, yet the majority remained near their original positions and no circular motion developed (Figure 4B; Videos S10 and S11).

Third, we tested white‐light responses using a Lumitec lamp (22 W, 6000 K). When oriented parallel to the ground and left undisturbed for 10 min, many isopods aggregated directly around the light source, but circular motion did not occur (Figure 4C; Videos S12–S14). However, when the same white light was positioned on a tripod perpendicular to the ground for 10 min, a mass migration toward the light ensued, followed by gradual assembly into organized circular movement beneath the light source (Figure 4D; Videos S15–S17). Across all three independent trials using a perpendicular white‐light source, we consistently induced large‐scale circular motion, replicating the naturally observed phenomenon.

### 3.3 Isopods Perform a Mass Circular Movement

**FIGURE 5:**
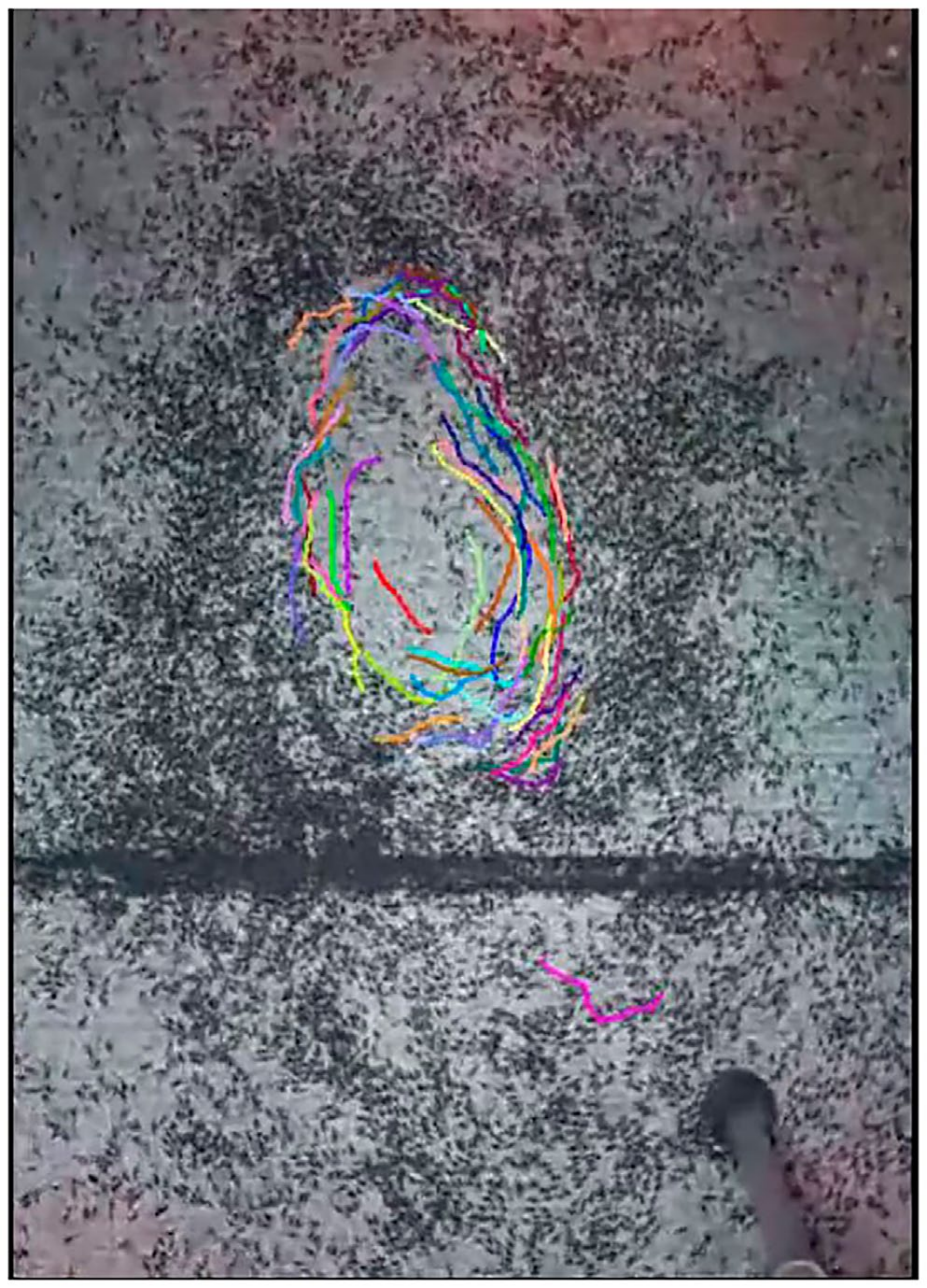
Tracking the movement of 60 isopods in Video S16, showing a collective circular pattern of movement.

Further examination of Video S16 revealed approximately 5575 isopods within the analyzed area, with a mean image size of 4.51 pixels (Table S1). To study their movement patterns, we manually tracked 60 clearly distinguishable individuals. The resulting trajectories revealed a pronounced circular motion (Figure 5; Video S18). Although the video was not long enough to capture full trajectories of individual isopods, the combined paths clearly show a coordinated, collective circular flow.

## 4 Discussion

Previous reports of mass isopod activity have been largely anecdotal, typically describing directional migrations rather than circular motion (Warburg et al. 1984; Warburg 1993). In contrast, our study documents a previously undescribed phenomenon—large‐scale circular movement of thousands of *A. sordidus* isopods—observed repeatedly at night, during summer, in northern Israel. The recurrence across different years and sites suggests that the behavior is both consistent and environmentally driven.

Quantitative analyses of a video documenting this behavior provided further insights into the dynamics of this behavior. Counting indicated approximately 5575 individuals within the defined circular area. This is surely an underestimate as many individuals were too tightly packed to be counted, whereas many others were outside the frame being analyzed. We estimate the actual number to be at least threefold higher than what we counted. Manual tracking of 60 individuals revealed coherent circular trajectories (Figure 5; Video S18). Although tracking was limited by video duration, the aggregate data strongly suggest coordinated collective motion, rather than random or density‐dependent movement. This finding supports the interpretation of the behavior as a true emergent group phenomenon.

The observed sex ratio of roughly 1:4 (males to females) corresponds with values reported for other *Armadillo* species (Warburg et al. 1984; AlJetlawi and Nair 1994). The high proportion of gravid females indicates that this aggregation is not related to breeding activity but may serve another ecological or behavioral function.

### 4.1 Attraction to Artificial Light and Circular Motion Formation

Attraction to ALAN is well documented across diverse terrestrial arthropods, including moths, beetles, flies, and millipedes (Owens and Lewis 2018; Foster et al. 2021; Davies et al. 2012; Gaston et al. 2013). Multiple hypotheses have been proposed to explain this phenomenon, including disruption of celestial navigation cues (the “transverse orientation” or “flight‐to‐light” hypothesis), positive phototaxis for mate finding or resource location, and disorientation caused by supernormal stimuli that override natural light‐avoidance behaviors (Owens and Lewis 2018; Fabian et al. 2024). While most studies focus on flying insects, recent work has shown that ALAN also affects ground‐dwelling arthropods such as beetles and woodlice, altering activity patterns, boldness, and aggregation behavior (Shellard and Mayor 2020; Owens and Lewis 2018; Dissegna and Chiandetti 2025).

In our study, isopods were strongly attracted to white light but not to UV light, and circular motion developed exclusively under specific lighting conditions. Critically, when white light was placed parallel to the ground (horizontal beam), isopods aggregated around the source but did not form circular motion (Figure 4C; Videos S12–S14). However, when the same light was positioned perpendicular to the ground (vertical beam), circular motion consistently emerged (Figure 4D; Videos S15–S17). This distinction indicates that the geometry of the illuminated area—rather than population size alone—plays a critical role in triggering circular motion. A vertically oriented light creates a roughly circular illuminated zone on the ground, and isopods appear to walk along the boundary of this lit area, producing the observed circular trajectory.

**FIGURE 6:**
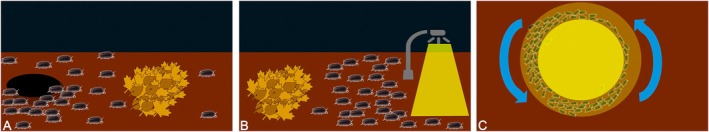
Proposed mechanism for isopod aggregation and circular movement. (A) Isopods emerge from shelters and forage. (B) Isopods are disturbed by light and are attracted to it. (C) Isopods move in collective circular motion around the boundaries of the light.

We therefore propose the following mechanism (Figure 6): At night, isopods emerge from shelters to forage and are attracted to nearby artificial light sources. Once density beneath a vertically oriented light source exceeds a threshold, individuals begin to follow the circular boundary of the illuminated zone, generating coherent circular motion. The threshold number of individuals required for circular motion likely scales with the size of the illuminated area—smaller light circles require fewer individuals, and larger circles require more. However, we acknowledge that the shape of illumination is the primary determinant: a circular lit area facilitates circular motion, whereas a horizontal beam does not, regardless of density. This geometric constraint may explain why circular motion was not observed in all field videos—variation in streetlight angle, intensity, or surrounding topography may have disrupted the formation of a circular photic boundary.

Other arthropods, including a *Scolopendra cingulata* centipede and *Porcellio laevis* woodlice, were observed in close proximity to the aggregations. In one instance, a *S. cingulata* individual appeared to prey upon the isopods, suggesting that ALAN‐induced aggregations may have ecological consequences beyond behavioral disruption.

In conclusion, our observations constitute the first formal documentation of mass aggregation and circular movement in isopods. This behavior was observed exclusively under artificial illumination, suggesting a strong anthropogenic influence driven by light pollution. The circular motion appears to result from the interaction between phototactic attraction and the geometry of the illuminated surface. Our observations seem to rule out the possibility of magnetic disorientation contributing to this behavior. Future research should investigate the sensory mechanisms underlying light attraction, test whether similar behaviors occur under natural light conditions (e.g., moonlight), explore why aggregations are restricted to a specific species and time of year, and assess the broader ecological impacts of ALAN on terrestrial isopod behavior and survival.

## Author Contributions

**Idan Sheizaf:** conceptualization (lead), formal analysis (lead), methodology (equal), writing – original draft (lead). **Eviatar Itzkovich:** investigation (lead), methodology (equal). **Ariel D. Chipman:** supervision (lead), writing – review and editing (equal).

## Funding

I.S. is supported by a Swiss National Science Foundation (SNSF) Sinergia award (grant number 198691).

## Conflicts of Interest

The authors declare no conflicts of interest.

## Supporting information


**Data S1:** ece373487‐sup‐0001‐DataS1.csv.

**Table S1:** Count of isopods per rectangular slice in the selected image. Columns representing count of isopods, the total area of isopods in the slice, average isopod size in slice, and percentage area of the isopods in the slice. Below the table, count summary of the isopods and their average size was calculated.

**Video S1:** Video captured on 5.7.2021 in Geva, Israel. Showing circular motion and aggregation of isopods.

**Video S2:** Video captured on 13.8.2023 in Eliad, Israel. Showing the same circular motion and aggregation pattern as Video S1.

**Video S3:** Video captured on 6.8.2025 in Eliad, Israel. Showing aggregation alone, without circular motion.

**Video S4:** Video captured on 11.8.2025 in Eliad, Israel. Showing circular motion and aggregation of isopods as seen before.

**Video S5:** Video captured on 16.8.2025 in Eliad, Israel. Showing the same circular motion and aggregation patterns as before.

**Videos S6–S7:** Videos showing experimental procedure of placing a magnet near the center of the circular movement phenomenon, 5 min after the beginning of the experiment.

**Videos S8–S9:** Videos showing experimental procedure of placing a UV light parallel to the ground, near the circular epicenter, 5 min after the beginning of the experiment.

**Videos S10–S11:** Videos showing experimental procedure of placing a UV light in perpendicular to the ground, near the circular epicenter, 5 min after the beginning of the experiment.

**Videos S12–S14:** Videos showing experimental procedure of placing a white light parallel to the ground, near the circular epicenter, 5 min after the beginning of the experiment.

**Videos S15–S17:** Videos showing experimental procedure of placing a white light in perpendicular to the ground, near the circular epicenter, 5 min after the beginning of the experiment.

**Video S18:** Video showing the tracking of the movement of 60 isopods in video 16, showing a collective circular pattern of movement.

**Text S1:** ece373487‐sup‐0014‐Text1.txt.

## Data Availability

All relevant data is included within the manuscript and Supporting Information.
